# Structural Variation of Chondroitin Sulfate Chains Contributes to the Molecular Heterogeneity of Perineuronal Nets

**DOI:** 10.3389/fnint.2018.00003

**Published:** 2018-02-02

**Authors:** Shinji Miyata, Satomi Nadanaka, Michihiro Igarashi, Hiroshi Kitagawa

**Affiliations:** ^1^Laboratory of Molecular Bioregulation, Graduate School of Bioagricultural Sciences, Nagoya University, Nagoya, Japan; ^2^Department of Biochemistry, Kobe Pharmaceutical University, Kobe, Japan; ^3^Department of Neurochemistry and Molecular Cell Biology, Graduate School of Medical and Dental Sciences and Trans-disciplinary Program, Niigata University, Niigata, Japan

**Keywords:** perineuronal nets, aggrecan, chondroitin sulfate, proteoglycan, parvalbumin-expressing neuron, Otx2, WFA

## Abstract

Aggrecan, a chondroitin sulfate (CS) proteoglycan, forms lattice-like extracellular matrix structures called perineuronal nets (PNNs). Neocortical PNNs primarily ensheath parvalbumin-expressing inhibitory neurons (parvalbumin, PV cells) late in brain development. Emerging evidence indicates that PNNs promote the maturation of PV cells by enhancing the incorporation of homeobox protein Otx2 and regulating experience-dependent neural plasticity. *Wisteria floribunda* agglutinin (WFA), an *N-acetylgalactosamine-specific* plant lectin, binds to the CS chains of aggrecan and has been widely used to visualize PNNs. Although PNNs show substantial molecular heterogeneity, the importance of this heterogeneity in neural plasticity remains unknown. Here, in addition to WFA lectin, we used the two monoclonal antibodies Cat315 and Cat316, both of which recognize the glycan structures of aggrecan, to investigate the molecular heterogeneity of PNNs. WFA detected the highest number of PNNs in all cortical layers, whereas Cat315 and Cat316 labeled only a subset of PNNs. WFA^+^, Cat315^+^, and Cat316^+^ PNNs showed different laminar distributions in the adult visual cortex. WFA, Cat315 and Cat316 detected distinct, but partially overlapping, populations of PNNs. Based on the reactivities of these probes, we categorized PNNs into four groups. We found that two subpopulation of PNNs, one with higher and one with lower WFA-staining are differentially labeled by Cat316 and Cat315, respectively. CS chains recognized by Cat316 were diminished in mice deficient in an enzyme involved in the initiation of CS-biosynthesis. Furthermore, WFA^+^ and Cat316^+^ aggrecan were spatially segregated and formed microdomains in a single PNN. Otx2 co-localized with Cat316^+^ but not with WFA^+^ aggrecan in PNNs. Our results suggest that the heterogeneity of PNNs around PV cells may affect the functional maturation of these cells.

## Introduction

Perineuronal nets (PNNs) are lattice-like extracellular matrix structures that surround synaptic contacts on the soma and the proximal dendrites of subpopulations of neurons (Celio et al., 1998). In the rodent visual cortex, the formation of PNNs occurs relatively late in postnatal development and reaches its maximum level at 10 weeks after birth, which parallels the decline in experience-dependent neural plasticity (Brückner et al., 2000; Pizzorusso et al., 2002). Recent studies have demonstrated that PNNs represent molecular brakes that restrict neural plasticity, and depletion of these structures enhances neural plasticity in many brain regions (Pizzorusso et al., 2002; Gogolla et al., 2009; Romberg et al., 2013; Happel et al., 2014; Xue et al., 2014). Chondroitin sulfate proteoglycans (CSPGs) belonging to the lectican family (aggrecan, versican, neurocan, and brevican) are major components of PNNs (Yamaguchi, 2000; Zimmermann and Dours-Zimmermann, 2008). Lecticans share structural domains, including an N-terminal globular domain, a long extended central region that contains covalently bound chondroitin sulfate (CS) chains, and a C-terminal globular domain. A N- and C-terminal globular domain bind to hyaluronan and tenascin-R, respectively, forming macromolecular aggregates in the extracellular space.

PNNs are highly heterogeneous in their molecular composition and in the glycan structures of their CSPGs. In many studies, the plant lectin *Wisteria floribunda* agglutinin (WFA), which shows preferential reactivity with glycans containing terminal *N*-acetylgalactosamine (GalNAc) residues, has been routinely used to visualize PNNs (Härtig et al., 1992; Haji-Ghassemi et al., 2016). CS chains are linear polysaccharides composed of a repeating disaccharide unit consisting of glucuronic acid (GlcA) and GalNAc. The repeating disaccharide units are modified with sulfates at different positions by a number of chondroitin sulfotransferases, which create large structural diversity in the CS chains (Mikami and Kitagawa, 2013; Miyata and Kitagawa, 2015, 2017). Previous studies have indicated that variation in the glycan structures on aggrecan contribute to the heterogeneity of PNNs (Lander et al., 1997; Matthews et al., 2002). Two monoclonal antibodies, Cat315 and Cat316, both of which react with aggrecan, label distinct but partially overlapping populations of PNNs. Cat315 recognizes the human natural killer-1 (HNK-1) glycan epitope on aggrecan (Matthews et al., 2002; Yabuno et al., 2015; Yamada et al., 2017). Both WFA and Cat316 are thought to bind to CS chains on aggrecan in PNNs (Brückner et al., 1998; Matthews et al., 2002; Giamanco et al., 2010). Primary cultured neurons from aggrecan-deficient mice lack staining for WFA, indicating that WFA recognizes CS chains carried by aggrecan (Giamanco et al., 2010). However, it is unclear whether WFA and Cat316 detect the same CS chain, because the precise glycan structures recognized by these probes have yet to be identified. Moreover, little is known about the functional significance of the heterogeneity of PNNs.

In several regions of the brain, including the cerebral cortex, PNNs are primarily formed around a single class of inhibitory interneurons expressing parvalbumin (PV cells; Kosaka and Heizmann, 1989; Härtig et al., 1992; Lüth et al., 1992). Emerging evidence indicates that PNNs affect PV cell function, which plays a central role in determining the timing of the critical period for experience-dependent plasticity. PNNs may actively modulate PV cell function by capturing secreted proteins at the cell surface (Bernard and Prochiantz, 2016; de Winter et al., 2016; Miyata and Kitagawa, 2017). One prominent example is a non-cell autonomous role of the Otx2 homeoprotein in experience-dependent plasticity (Sugiyama et al., 2008). Otx2 produced in the retina and choroid plexus is transported to PV cells in the cerebral cortex, where it promotes maturation of PV cells (Sugiyama et al., 2008; Spatazza et al., 2013; Lee et al., 2017). We previously showed that the incorporation of Otx2 into PV cells was affected by the sulfation pattern of the CS chains in PNNs: the accumulation of Otx2 in PV cells was reduced in transgenic mice overexpressing the juvenile-type CS sulfation pattern (Miyata et al., 2012). Using chemically synthesized CS oligosaccharides, other studies indicated that Otx2 had a high affinity for CS chains rich in disulfated disaccharide units (Beurdeley et al., 2012; Despras et al., 2013). Otx2 was co-immunoprecipitated with aggrecan in a manner dependent on the presence of CS chains (Hou et al., 2017). These data collectively suggest that Otx2 is sequestrated to PNNs via CS chains. However, the endogenous CS structure responsible for Otx2 binding remains undetermined. Here we report that structural variation of CS chains of aggrecan contributes to the molecular heterogeneity of PNNs. The heterogeneity of PNNs around PV cells may affect the functional maturation of these cells via the selective binding of Otx2 to specific CS chains.

## Materials and Methods

### Animals

Transgenic mice overexpressing human chondroitin 6-sulfotransferase-1 (C6ST-1 TG) and knockout mice for chondroitin *N*-acetylgalactosaminyltransferase-1 (ChGn-1 KO) were described previously (Watanabe et al., 2010; Miyata et al., 2012). Both strains are on the C57BL/6 background. Three-months-old adult male C57BL/6 mice were used in this study. All animal experimental studies were conducted with the approval of the Animal Care and Use Committee of Kobe Pharmaceutical University and the Nagoya University Animal Care Committee.

### Histochemistry

Mice were perfused transcardially with PBS followed by 4% paraformaldehyde in PBS. Brains were removed and post-fixed overnight with 4% paraformaldehyde in PBS. Coronal sections (50 mm thick) were cut with a vibratome (LEICA). Sections were permeabilized with 0.2% Triton X-100 in PBS for 20 min, blocked with 2% BSA in PBS for 1 h, and incubated overnight at room temperature with the primary antibodies described in Table 1. Sections were incubated with the appropriate Alexa488/594/647-labeled secondary antibodies (Invitrogen) for 1 h at room temperature. For WFA lectin staining, sections were incubated with biotinylated WFA followed by secondary labeling with Alexa488/647-conjugated streptavidin. For enzymatic digestions, the permeabilized sections were digested with 5 milliunits of chondroitinase ABC (Seikagaku Corporation, Japan) or 5 units of β-*N*-acetylhexosaminidase (New England Biolabs, Ipswich, MA, USA) for 4 h at 37°C before incubation with the primary antibodies.

**Table 1:** List of the antibodies used in this study.

Antibody	Isotype	Source	Dilution
Cat315	Mouse IgM	Millipore, MAB1581	1:2000
Cat316	Mouse IgM	Millipore, MAB1582	1:2000
Anti-PV	Mouse IgG1	Swant, PV235	1:2000
Anti-Otx2	Goat IgG	Santa Cruz Bio, sc-30659	1:50
WFA	Lectin	EY Laboratories, BA-3101-1	1:2000

### Image Analysis

Images were captured with an FV1200 laser scanning confocal microscope (OLYMPUS). For quantification of the number of PNNs, labeled cells were counted in 1.2–1.3 mm area spanning all cortical layers of the primary visual cortex. For fluorescence intensity analysis, the exposure time, gain and offset were set to ensure a high signal but to avoid saturation. A region of interest (ROI) circumscribing single PNN was manually traced, and the average signal intensity within a ROI was measured using FV10 ASW software (OLYMPUS). For the three-dimensional reconstruction of PNNs, 12-15 Z-stack images at 0.46 μm intervals covering approximately 5.5–7 μm in depth were acquired using a 100× objective and processed using FV10 ASW software (OLYMPUS).

### Immunoblotting

Brains were homogenized with a tight-fitting Potter glass homogenizer in PBS buffer containing 1% Triton X-100 and protease inhibitor cocktail and incubated on ice for 60 min. After centrifugation at 15,000 rpm for 30 min at 4°C, the protein concentrations of the supernatants were determined using a BCA assay kit (Thermo). For chondroitinase digestion, the brain lysate (200 μg as protein) was digested with 5 milliunits of chondroitinase ABC (Seikagaku Corporation, Japan) for 2 h at 37°C. Undigested or chondroitinase-digested lysate (20 μg as protein) was separated by 5% acrylamide gel electrophoresis, transferred onto a PVDF membranes (GE Healthcare), and incubated overnight at 4°C with Cat316 antibody (Millipore, rabbit polyclonal IgG, 1:10,000). The blots were subsequently incubated with the appropriate HRP-labeled secondary antibodies for 1 h at room temperature and developed using an ECL detection system (GE Healthcare).

### Preparation and Biotinylation of CS Chains from Mouse Brain

Brains were homogenized in ice-cold PBS containing protease inhibitor cocktail and treated with 0.5 M LiOH at 4°C overnight to liberate *O*-linked saccharides from the core protein (Izumikawa et al., 2015). After centrifugation, the supernatant was applied to a column of anion-exchange resin AG 50W-X2 (H+ form, Bio-Rad) equilibrated with H_2_O. The flow-through fraction containing the liberated *O-linked* saccharides was neutralized with 1 M NH_4_HCO_3_ and subjected to gel filtration column chromatography (PD-10, GE Healthcare). The flow-through fraction was collected, evaporated to dryness and dissolved in water. An aliquot of the sample was digested with chondroitinase ABC (Seikagaku Corporation, Japan) and derivatized with a fluorophore, 2-aminobenzamide. The derivatized unsaturated disaccharides were analyzed by anion-exchange HPLC (SLC-10A, Shimadzu) using a PA-03 column (YMC Co.) and quantified as described previously (Kitagawa et al., 1995).

The liberated *O*-linked saccharides were biotinylated as described previously (Deepa et al., 2002). Briefly, the liberated *O*-linked saccharides fraction (1.2 mg as total CS disaccharides) was dissolved in 100 mM MES, pH 5.5. The solution was mixed with a 50 mM solution of freshly prepared biotin-LC-hydrazide (Pierce) in dimethyl sulfoxide. EDAC (1-(3-Dimethylaminopropyl)-3-ethylcarbodiimide) hydrochloride was added to this mixture and incubated for 2 h at room temperature by gently mixing the solution. The reaction mixture was subjected to gel filtration column chromatography (PD-10), and the flow-through fraction was collected.

### Enzyme Linked Immunosorbent Assays (ELISA)

Biotinylated *O*-linked saccharides (1–100 ng as total CS disaccharides) were immobilized on a 96-well BD BioCoat streptavidin assay plate (BD Biosciences; Deepa et al., 2002). After blocking with 2% BSA in PBS, Cat316 antibody (diluted 20,000-fold with 2% BSA in PBS) was added to each well and incubated at 4°C overnight. After washing with PBS, peroxidase-conjugated anti-mouse IgG+M (1/10,000 in 2% BSA/PBS) was added to the wells and incubated for 2 h at room temperature. After washing, the color was developed using a ABTS (2,2′-azino-bis(3-ethylbenzthiazoline-6-sulfonic acid)) substrate kit (Vector Laboratories) and the optical density was measured at 415 nm using a microplate reader (Bio-Rad model 550).

### Statistical Analysis

Statistical significance was determined using the unpaired two-tailed Student’s *t*-test. Differences were considered significant at a *P* value of less than 0.05.

## Results

### Laminar Distributions of WFA^+^, Cat315^+^, and Cat316^+^ PNNs in the Primary Visual Cortex of Adult Mice

**Figure 1:**
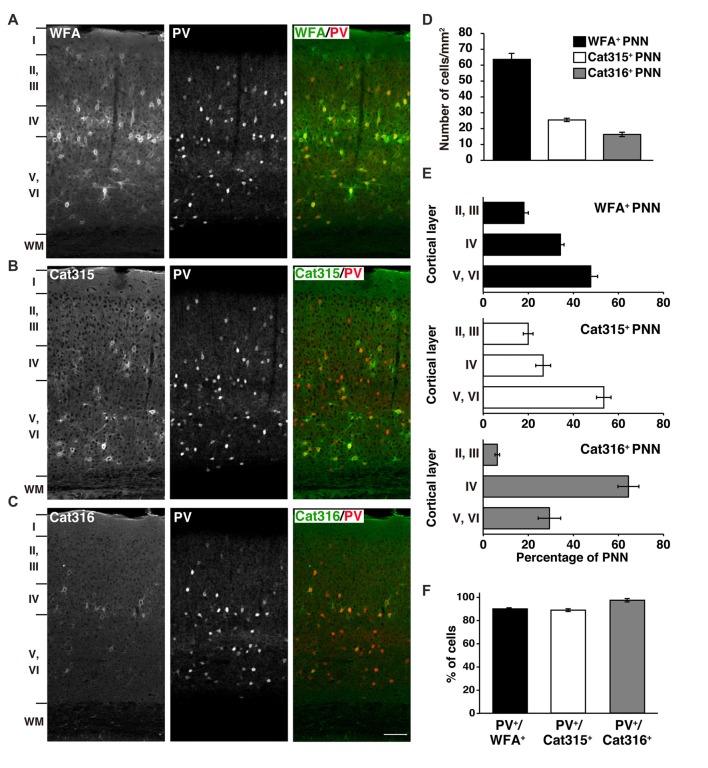
Different laminar distributions of WFA^+^, Cat315^+^ and Cat316^+^ perineuronal nets (PNNs) in the adult visual cortex. **(A–C)** Immunohistochemical detection of different PNNs in the primary visual cortex of 3-months-old adult mouse using *Wisteria floribunda* agglutinin (WFA) **(A)**, Cat315 **(B)**, and Cat316 **(C)**. WFA-staining was also observed in the neuropil in layer IV. Cortical layers are indicated in Roman numerals to the left of the panels. WM, white matter. Scale bar, 50 μm. **(D)** The numbers of WFA^+^ (closed bar), Cat315^+^ (open bar) and Cat316^+^ (gray bar) PNNs. WFA detected the highest number of PNNs. **(E)** Laminar distributions of PNNs. Bar graphs represent percentage of WFA^+^ (upper), Cat315^+^ (middle) and Cat316^+^ (lower) PNNs in each cortical layer. Cat315^+^ PNNs were most abundant in layers V and VI, whereas Cat316^+^ PNNs were concentrated in layer IV. **(F)** Percentage of WFA^+^ (closed bar), Cat315^+^ (open bar), and Cat316^+^ (gray bar) PNNs formed around parvalbumin cells (PV cells). *n* = 327, 131 and 80 cells from three mice for WFA^+^, Cat315^+^ and Cat316^+^ PNNs, respectively. Error bars represent SEM.

Using WFA lectin and Cat315/Cat316 antibodies, we investigated the molecular heterogeneity of PNNs in the mouse primary visual cortex, where the involvement of PNNs in experience-dependent neural plasticity has been repeatedly reported (Carulli et al., 2010; Beurdeley et al., 2012; Miyata et al., 2012; Hou et al., 2017). Among the three probes, WFA detected the highest number of PNNs in all cortical layers, except layer I (Figures 1A,D). Cat315 and Cat316 immunoreactivities were observed around a subset of neurons, as previously described in the cat and rat visual cortex (Figures 1B,C; Lander et al., 1997; Matthews et al., 2002). We found that the number of PNNs stained by Cat315 or Cat316 was markedly lower than that of WFA^+^ PNNs (Figure 1D). The laminar distributions of PNNs detected by these probes were different: Cat315^+^ PNNs were most abundant in layers V and VI, whereas Cat316^+^ PNNs were concentrated in layer IV (Figure 1E). We examined which neuronal subtypes are enwrapped by these PNNs. Most neurons surrounded by WFA^+^ PNNs were PV^+^ neurons (90.1 ± 0.9%), consistent with previous studies (Figure 1F). We found that 89.0 ± 1.2% of Cat315^+^ PNNs and 97.8 ± 1.4% of Cat316^+^ PNNs were formed around PV cells. In contrast, only a small population of PV cells was surrounded by Cat315^+^ or Cat316^+^ PNNs, indicating that Cat315 and Cat316 each selectively label a subset of PV cells in the adult mouse visual cortex.

### Identification of Four Types of PNNs, Which Differ in the Glycan Structures of Aggrecan

**Figure 2:**
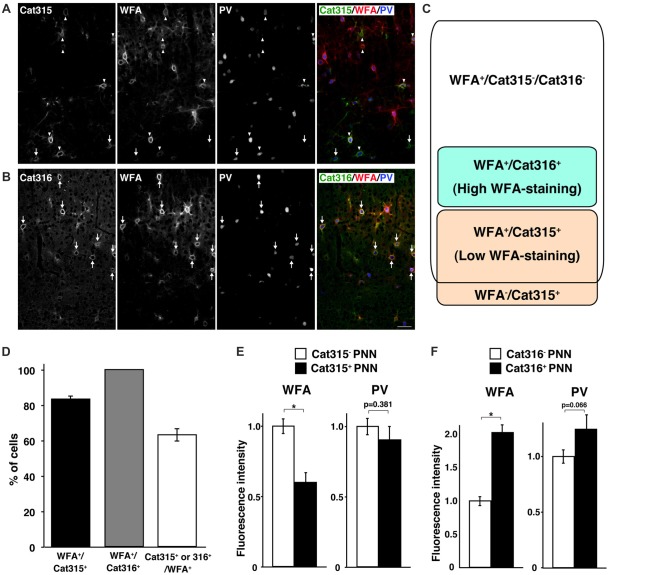
Identification of four types of PNNs. **(A,B)** Triple staining of WFA (red), PV (blue) and Cat315 (green in **A**) or Cat316 (green in **B**). Arrowheads and arrows in **(A)** indicate WFA^+^/Cat315^+^ and WFA^−^/Cat315^+^ PNNs, respectively. Arrows in **(B)** indicate WFA^+^/Cat316^+^ PNNs, Scale bar, 50 μm. **(C)** Four types of PNNs, which differ in the glycan structures of aggrecan. **(D)** Percentage of WFA^+^/Cat315^+^ (closed bar), WFA^+^/Cat316^+^ (gray and Cat315^+^ or Cat316^+^/WFA^+^ (open bar) PNNs (*n* = 131, 80 and 327 cells, respectively). **(E)** Fluorescence intensity of WFA-staining (left panels) and PV-staining (right panels) in Cat315^+^ PNNs (closed bar) relative to Cat315^−^ PNNs (closed bar). *n* = 62 and 52 cells from three mice for Cat315^−^ and Cat315^+^ PNNs, respectively. **(F)** Fluorescence intensity of WFA-staining and PV-staining in Cat316^+^ PNNs (closed bar) relative to Cat316^−^ PNNs (closed bar). *n* = 72 and 55 cells from three mice for Cat316^−^ and Cat316^+^ PNNs, respectively. Asterisks denote significant differences (*P* < 0.00001, Student’s *t*-test) between two groups. Error bars represent SEM.

WFA has been the most widely used lectin for visualizing PNNs in previous reports. Thus, we next investigated whether WFA^+^ PNNs overlap with Cat315^+^ or Cat316^+^ PNNs. Double staining with WFA and Cat315 showed that the majority of Cat315^+^ PNNs (83.5 ± 1.8%) were also positive for WFA, and a small population of Cat315^+^ PNNs (16.5 ± 1.8%) was not labeled by WFA (Figures 2A,D). We found that essentially all Cat316^+^ PNNs were also positive for WFA (Figures 2B,D). Conversely, 63.4 ± 3.5% of WFA^+^ PNNs were positive for either Cat315 or Cat316 (Figure 2D). Based on these data, we categorized PNNs into four groups: (i) WFA^+^/Cat315^−^/Cat316^−^ PNNs; (ii) WFA^+^/Cat315^+^ PNNs; (iii) WFA^−^/Cat315^+^ PNNs; and (iv) WFA^+^/Cat316^+^ PNNs (Figure 2C). Because both Cat315 and Cat316 are mouse IgM antibodies, it was difficult to perform double staining, and we could not address whether there are any PNNs positive for both Cat315 and Cat316. The detection of WFA^−^/Cat315^+^ PNNs raises the possibility that the reactivity of Cat315 may be negatively correlated with that of WFA. To test this possibility, we compared the fluorescence intensity of WFA-staining between Cat315^+^ and Cat315^−^ PNNs. Cat315^+^ PNNs showed a significantly lower intensity of WFA-staining than Cat315^−^ PNNs, implying that the expression of Cat315 epitope may repress the expression of CS chains detected by WFA (Figure 2E; see “Discussion” section). We found that the weaker WFA-staining in Cat315^+^ PNNs was not due to competitive binding between WFA and Cat315, because pre-incubation with WFA did not affect Cat315-staining (data not shown). In stark contrast to Cat315^+^ PNNs, Cat316^+^ PNNs showed a significantly higher intensity of WFA-staining compared with Cat316^−^ PNNs (Figure 2F). These results indicate that a subpopulation of PNNs with higher or lower WFA-staining is differentially labeled by Cat316 or Cat315, respectively. The expression level of PV has been shown to correlate with the functional maturation of PV cells. No difference was observed in the fluorescence intensity of PV-staining between Cat315^+^ and Cat315^−^ PNNs (Figure 2E). In contrast, PV-staining was higher in Cat316^+^ PNNs than in Cat316^−^ PNNs with a trend towards significance (*p* = 0.066; Figure 2F).

### Otx2 Co-localizes with Cat-316^+^ CS Chains in PNNs

**Figure 3:**
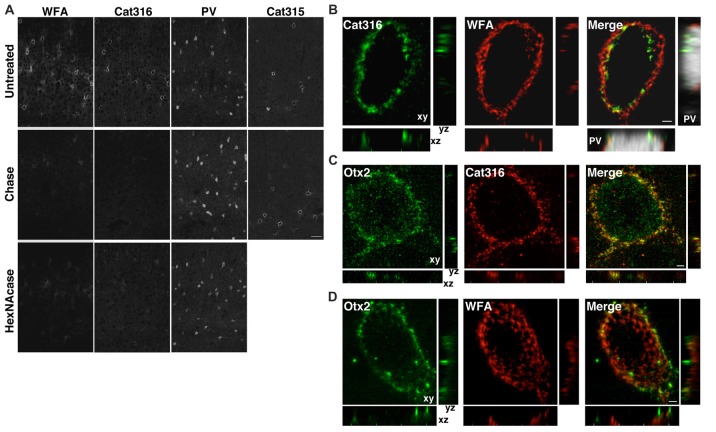
WFA^+^ and Cat316^+^ chondroitin sulfate (CS) chains form microdomains in PNNs. **(A)** Enzymatic digestion of PNNs. The reactivities of WFA and Cat316, but not Cat315 and PV, were diminished by treatment with chondroitinase ABC (Chase). WFA- and Cat316-staining were also markedly decreased upon treatment with β-*N*-acetylhexosaminidase (HexNAcase). Scale bar, 50 μm. **(B)** The three-dimensional reconstruction showed a meshwork of WFA^+^ CS chains (red). Cat316^+^ CS chains (green) showed a punctate pattern and only partially overlapped with WFA^+^ CS chains. Orthogonal views of the XZ plane (bottom panel) and YZ plane (right panel) revealed distinct distribution patterns of WFA^+^ and Cat316^+^ CS chains on the surface of PV cell (gray). **(C,D)** Otx2 (green) co-localized with Cat316^+^ (red in **C**), but not with WFA^+^ CS chains (red in **D**). Scale bar, 2 μm.

Both WFA^+^ and Cat316^+^ PNNs disappeared upon treatment with chondroitinase ABC, which digests CS chains on CSPGs, confirming that these probes recognize CS chains in PNNs, as previously reported (Figure 3A; Matthews et al., 2002; Pizzorusso et al., 2002). In contrast, the immunoreactivity of Cat315 was unaffected by chondroitinase ABC. We found that WFA bound to the nonreducing terminal GalNAc residues of CS chains in PNNs, as determined by the observation that WFA-staining was lost upon treatment with β-*N*-acetylhexosaminidase, which cleaves the terminal GalNAc and *N*-acetylgalactosamine (GlcNAc) residues of glycan chains (Figure 3A). The reactivity of Cat316 was also removed by β-*N*-acetylhexosaminidase, indicating that the epitope of Cat316 contains the nonreducing terminal GalNAc residues of the CS chains. Given that β-*N*-acetylhexosaminidase from *Streptomyces plicatus* liberates both non-sulfated and sulfated GalNAc residues (Kulik et al., 2015), it is not clear whether sulfation of the GalNAc residue affects recognition by these probes. However, because only a subset of WFA^+^ PNN overlaps with Cat316^+^ PNNs, WFA and Cat316 likely recognize different CS chains.

Although recent studies have proposed that the CS chains in PNNs sequestrate Otx2 and accelerate the functional maturation of PV cells (Beurdeley et al., 2012; Despras et al., 2013; Lee et al., 2017), the endogenous CS structure responsible for Otx2 binding remains unclear. We found that WFA^+^ and Cat316^+^ CS chains were spatially segregated and formed microdomains in a single WFA^+^/Cat316^+^ PNN (Figure 3B). The three-dimensional reconstruction of WFA^+^ CS chains revealed a distinct meshwork surrounding the soma and proximal dendrites of PV cells. Cat316^+^ CS chains were distributed in a punctate pattern rather than in a meshwork structure, and only partially overlapped with WFA^+^ CS chains. Prominent extracellular accumulation of Otx2 was observed in WFA^+^/Cat316^+^ PNNs, particularly in layer IV in the adult visual cortex (Figure 3C). Notably, Otx2 co-localized with Cat316^+^, but not with WFA^+^ CS chains in WFA^+^/Cat316^+^ PNNs (Figures 3C,D), suggesting the selective binding of Otx2 to Cat316^+^ CS chains.

### Reduced Expression of Cat316^+^ CS Chains in ChGn-1 KO Mice

**Figure 4:**
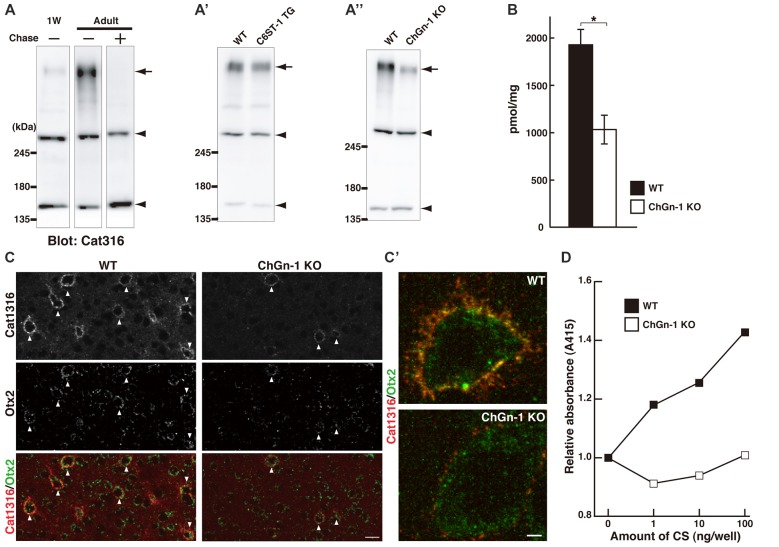
Reduced level of Cat316^+^ CS chains in ChGn-1 KO mice. **(A)** Expression of Cat316^+^ CS chains in the mouse brain increased during postnatal development. Brain lysates prepared from 1-week-old (1W) and 3-month-old (Adult) mice were detected by Cat316. The reactivity was lost after treatment with chondroitinase ABC (Chase) as indicated by an arrow. Arrowheads indicate chondroitinase ABC-insensitive non-specific bands. **(A′,A″)** Cat316^+^ CS chains were markedly diminished in the brain of ChGn-1 KO **(A″)**, but not in C6ST-1 TG mice **(A′)**. Data are representative of at least two mice per group. **(B)** Quantification of total amount of CS chains in the cerebrum of wild-type (WT; closed bar) and ChGn-1 KO mice (open bar). Asterisks denote significant differences (*P* < 0.05, Student’s *t*-test) between two groups. Error bars represent SEM. *n* = 3 mice for each group. **(C)** Immunohistochemical detection of Cat316^+^ PNNs (red) and Otx2 (green) in the adult brain of WT and ChGn-1 KO mice. Arrowheads indicate co-localization of Cat316^+^ PNNs and Otx2. Magnified views are shown in **(C′)**. Scale bars, 50 μm in **(C)** and 2 μm in **(C′)**. **(D)** CS chains isolated from WT (closed square) and ChGn-1 KO (open square) mice were biotinylated and immobilized on a streptavidin-coated plate (1–100 ng/well). Wells were incubated with Cat316 antibody, followed by peroxidase-conjugated secondary antibody. The color was developed using ABTS substrate kit and the optical density was measured at 415 nm. Absorbance was expressed as relative values to control (0 ng/well of CS). Data are means of duplicate wells and are representative of two independent experiments.

Our results to date suggest that Cat316^+^ CS chains may be important for the accumulation of Otx2. Western blot analysis showed that the expression level of Cat316^+^ CS chains increased during postnatal development in wild-type (WT) mice, and that reactivity was lost after treatment with chondroitinase ABC (Figure 4A). We previously reported that C6ST-1 TG mice, which overexpress the 6-sulfated disaccharide unit (GlcA-GalNAc(6-sulfate)), exhibited impaired formation of WFA^+^ PNNs and decreased accumulation of Otx2 (Miyata et al., 2012; Miyata and Kitagawa, 2016a). However, the present study showed no obvious change in the Cat316 immunoreactivity in C6ST-1 TG mice compared with WT mice (Figure 4A′), indicating that the expression of Cat316^+^ CS chains is not affected by 6-sulfated disaccharide unit.

It was recently reported that both the formation of WFA^+^ PNNs and the accumulation of Otx2 was reduced in mice lacking ChGn-1, which is involved in the initiation of CS biosynthesis (Gotoh et al., 2002; Uyama et al., 2002; Hou et al., 2017). Biochemical quantification of CS chains isolated from the adult cerebrum revealed that the total amount of CS chains was reduced by approximately half in ChGn-1 KO mice compared with WT mice (Figure 4B). This suggests that one-half of the CS chains in the adult cerebrum are synthesized in a ChGn-1-dependent manner, and that other glycosyltransferases, such as ChGn-2, may be responsible for the synthesis of the remaining CS chains. In the adult WT cerebrum, CS chains were mostly composed of the 4-sulfated disaccharide unit (GlcA-GalNAc(4-sulfate)), with the non-sulfated disaccharide unit (GlcA-GalNAc) accounting for 9% of the total CS (Table 2). ChGn-1 KO mice showed a slightly, but statistically significant, decreased proportion of 4-sulfated disaccharide unit and an increased proportion of non-sulfated disaccharide unit relative to WT mice. Although the disulfated disaccharide units (GlcA-GalNAc(4,6-disulfate) and GlcA(2-sulfate)-GalNAc(6-sulfate)) were minor components in the adult cerebrum, their proportions were significantly different between the two groups. Notably, we found that Cat316^+^ CS chains were markedly diminished in the brain of ChGn-1 KO mice (Figure 4A″). Furthermore, ChGn-1 KO mice showed a decreased number of Cat316^+^ PNNs, accompanied with reduced accumulation of Otx2 (Figures 4C,C′). The reduced reactivity of Cat316 may result from a global reduction of CS chains or selective deletion of Cat316^+^ CS chains in ChGn-1 KO mice. To distinguish between these possibilities, CS chains were liberated from core proteins, partially purified and assessed for reactivity against Cat316 by Enzyme linked immunosorbent assays (ELISA). CS chains isolated from WT mouse brain showed a dose-dependent increase in Cat316 reactivity (Figure 4D). When an equal amount of CS chains was immobilized, the reactivity of CS chains from ChGn-1 KO mice was still lower than that of WT mice, suggesting that Cat316 recognizes a specific CS structure that is selectively reduced in the absence of ChGn-1.

**Table 2:** Disaccharide composition of CS chains in WT and ChGn-1 KO cerebrum.

	Unsaturated disaccharide^a^ (mol%)^b^				
	∆Di-0S	∆Di-6S	∆Di-4S	∆Di-diS_D_	∆Di-diS_E_
WT	9.1 ± 0.19	1.8 ± 0.27	87.2 ± 0.14	0.7 ± 0.05	1.2 ± 0.05
ChGn-1 KO	12.1 ± 1.04*	1.8 ± 0.07	84.0 ± 0.93*	0.4 ± 0.02*	1.8 ± 0.05*

*^a^ΔDi-0S, Δ^4,5^HexUAα1–3GalNAc; ΔDi-6S, Δ^4,5^HexUAα1–3GalNAc(6-O-sulfate); ΔDi-4S, Δ^4,5^HexUAα1–3GalNAc(4-O-sulfate); ΔDi-diS_D_, Δ^4,5^HexUA(2-O-sulfate)α1–3Gal-NAc(6-O-sulfate); ∆Di-diS_E_, Δ^4,5^HexUAα1–3GalNAc(4,6-O-disulfate). ^b^The values (mean ± SEM) are expressed as mol percentage of total CS disaccharides (*n* = 3). Asterisks denote significant differences (P < 0.05, Student’s t-test) between WT vs. ChGn-1 KO*.

## Discussion

Heterogeneity of PNNs may arise from variation in the molecular composition and/or differences in the glycan structure of CSPGs. For example, by combining colloidal iron hydroxide staining for detection of polyanionic components with GalNAc-binding lectins such as WFA, heterogeneous PNNs were reported in several brain regions (Seeger et al., 1994). In this study, we identified four types of PNNs that differ in the glycan structures of aggrecan: (i) WFA^+^/Cat315^−^/Cat316^−^ PNNs; (ii) WFA^+^/Cat315^+^ PNNs; (iii) WFA^−^/Cat315^+^ PNNs; and (iv) WFA^+^/Cat316^+^ PNNs. These four types of PNNs primarily surrounded PV cells in the mouse visual cortex. Prominent extracellular accumulation of Otx2 was observed in WFA^+^/Cat316^+^ PNNs. Notably, WFA^+^ and Cat-316^+^ CS chains were spatially segregated in a single PNN, and Otx2 co-localized with Cat-316^+^, but not with WFA^+^ CS chains in PNNs. These results suggest that the heterogeneity of PNNs affects the localization of Otx2, and thereby controls the functional maturation of PV cells. Recent studies reported that distributions of aggrecan and brevican in a single PNN are different: aggrecan surrounds the synaptic contacts, whereas brevican is localized at the synaptic cleft (Blosa et al., 2013, 2015; Oohashi et al., 2015). Condensation of aggrecan into PNNs is not affected in brevican-deficient mice, suggesting that the localization of these two CSPGs in PNNs is independently regulated. In this study, we showed that aggrecan glycoforms having different glycan structures form microdomains in PNN. Therefore, both core proteins and glycan chains may be key factors determining the precise localization of CSPGs in PNNs.

**Figure 5:**
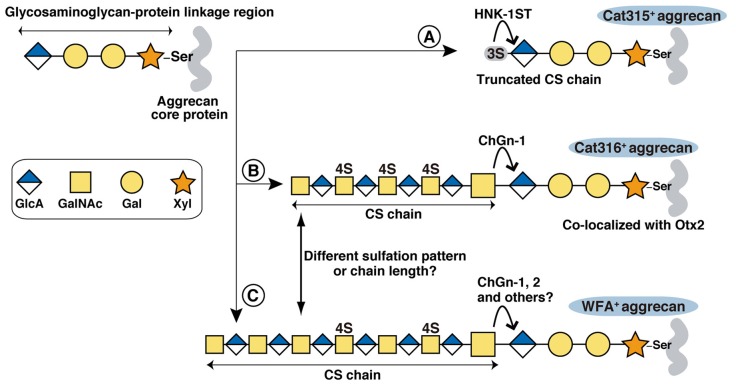
Proposed biosynthetic pathways of Cat315^+^, Cat316^+^ and WFA^+^ aggrecan. The glycosaminoglycan-protein linkage region is assembled on specific Ser residues of aggrecan. Although aggrecan core protein contains more than 100 potential CS attachment sites, only one attachment site is shown in the figure for simplicity. **(A)** HNK-1ST transfer a sulfate to the non-reducing terminal GlcA residue of the linkage region, generating a Cat315^+^ truncated CS chain that prevents further elongation of the repeating disaccharides. **(B,C)** Transfer of a GalNAc to the terminal GlcA residue in the linkage region by ChGn-1, 2 or chondroitin polymerases triggers the elongation of a CS chain. Cat316^+^ CS chains are mainly produced by a ChGn-1-dependent pathway **(B)**. WFA^+^ and Cat316^+^ CS chains may have different sulfation pattern and/or chain length **(C)**. Otx2 may selectively bind to Cat316^+^ CS chains.

Although both WFA and Cat316 reportedly bind to CS chains on aggrecan (Matthews et al., 2002; Giamanco et al., 2010), our results indicate that WFA and Cat316 recognize different types of CS chains. We previously reported that in the visual cortex staining with antibody recognizing core protein portion of aggrecan showed a well-formed meshwork and a similar pattern to that with WFA (Miyata et al., 2012; Miyata and Kitagawa, 2016a). By contrast, in the stratum oriens of the hippocampus CA3 region, only 10%–20% of aggrecan-positive PNNs are co-stained with WFA (Yamada and Jinno, 2017), suggesting different CS modifications on aggrecan among different brain regions. WFA has specificity for the terminal GalNAc residues on glycan chains, whereas the glycan structure recognized by Cat316 remains undetermined. Our study revealed that CS chains isolated from the brain of ChGn-1 KO mice showed decreased reactivity with Cat316. Usually, CS chains are attached to specific serine (Ser) residues of the core protein via the glycosaminoglycan-protein linkage region (GlcAβ1-3Galβ1-3Galβ1-4Xylβ1-*O*-Ser), where Gal and Xyl represent galactose and xylose, respectively (Figure 5). ChGn-1 and -2 transfer the first GalNAc residue to the non-reducing terminal GlcA residue of the linkage region (Gotoh et al., 2002; Uyama et al., 2002, 2003; Sato et al., 2003; Mikami and Kitagawa, 2013). This step allows subsequent elongation of the CS chains. The marked reduction of Cat316^+^ CS chains in ChGn-1 KO mice indicates that Cat316^+^ CS chains are mainly produced by a ChGn-1-dependent biosynthetic pathway. In cartilage, ChGn-1 is required for the production of specific short-chain CS species (Watanabe et al., 2010), implying that Cat316 may recognize short-chain CS species in the brain. During preparation of our manuscript, a recent report showed that Cat316 preferentially bind to CS chains rich in 4-sulfated disaccharide unit, indicating that Cat316 may recognize a 4-sulfation-related structure on aggrecan (Yang et al., 2017). Co-localization of Otx2 and Cat316^+^ CS chains raises the possibility that Otx2 may preferentially bind to Cat316^+^ CS chains. Using chemically synthesized CS oligosaccharides and CS polysaccharides derived from marine organisms, previous studies showed that Otx2 had a high affinity for CS chains rich in the 4,6-disulfated disaccharide unit (GlcA-GalNAc(4,6-sulfate); Beurdeley et al., 2012; Despras et al., 2013). Although Cat316^+^ CS chains and Otx2 accumulation were markedly reduced in ChGn-1-KO mice, the proportion of 4 6-disulfated disaccharide unit was not decreased, but rather increased in ChGn-1-KO mice. Further studies are required to elucidate the structural determinants responsible for Cat316 and Otx2 recognition.

We found that the intensity of WFA staining was significantly weaker in Cat315^+^ PNNs compared with Cat315^−^ PNNs. A recent study identified a sulfated glycosaminoglycan-protein linkage region (SO_3_-3GlcAβ1-3Galβ1-3Galβ1-4Xylβ1-*O*-Ser), on aggrecan as a novel type of HNK-1 epitope recognized by Cat315 (Yabuno et al., 2015; Figure 5). Sulfation of the GlcA residue in the linkage region prevents further elongation of the CS chains and reduces the amount of CS chains on aggrecan *in vitro*. Our present study provides additional *in vivo* evidence supporting that the HNK-1 epitope on the linkage region negatively regulates the expression of CS chains in PNNs. The formation of Cat315^+^ PNNs is dependent on neuronal activity both in the barrel cortex and the superior olivary complex as sensory deprivation markedly reduces the number of Cat315^+^ PNNs (McRae et al., 2007; Myers et al., 2012). Therefore, it is possible that activity-dependent expression of the linkage type HNK-1 epitope precisely controls the number of CS chains on aggrecan and thereby modulates the functional properties of PNNs.

Cat315^+^ PNNs and Cat316^+^ PNNs showed different laminar distributions in the visual cortex: Cat315^+^ PNNs are concentrated around PV cells located in layer V and VI, whereas Cat316^+^ PNNs are abundant around PV cells in layer IV. Thee laminar distribution of PV cells in the cerebral cortex correlates with birthdate: early-born PV cells primarily populate the deep layers (V and VI), while late-born PV cells colonize the upper layers (II, III and IV; Rymar and Sadikot, 2007; Bartolini et al., 2013). PV cells are heterogeneous not only in location but also in morphology, connectivity and electrophysiological characteristics. For example, early and late-born PV cells show distinct connectivities and roles in learning (Donato et al., 2013, 2015). Our results suggest that the expression levels of CS-synthesizing enzymes in PV cells may be responsible for the heterogeneity of PNNs. In this regard, Otx2 may act as a transcription factor that promotes formation of PNNs after being incorporated into PV cells. A recent ChIP-seq (chromatin immunoprecipitation sequencing) analysis found a strong Otx2 binding to the promoter region of the ChGn-1 gene (Sakai et al., 2017), suggesting that Otx2 may directly regulate expression of the ChGn-1 and Cat316^+^ PNNs, which in turn facilitates further accumulation of Otx2. On the other hand, the formation of Cat315^+^ PNNs may be controlled by expression of HNK-1 sulfotransferase (HNK-1ST), which transfers sulfate to the non-reducing terminal GlcA residue of the linkage region (Senn et al., 2002; Morise et al., 2017). Forced expression of HNK-1ST increases the amount of linkage type HNK-1 epitope on aggrecan *in vitro* (Yabuno et al., 2015). Since ChGn-1 and HNK-1ST compete for the same substrate (the non-reducing terminal GlcA residue of the linkage region), a balance in the expression and activities of these two enzymes could be a mechanism for creating the observed heterogeneity of PNNs (Figure 5). Thus, it will be important to understand how these different types of PNNs are generated during development and how they modulate PV cell function.

PNNs may capture not only Otx2, but also other secreted molecules, such as semaphorin3A and neuronal activity-regulated pentraxin to regulate PV cell function (Chang et al., 2010; Dick et al., 2013; de Winter et al., 2016). Comprehensive analysis of protein-protein and protein-glycan interactions among PNN molecules will provide new insight into the roles of distinct aggrecan glycoforms. Dysregulation of PNNs has been implicated in several neuronal disorders (Sorg et al., 2016). It was reported that the glycan structures of CSPGs were affected in schizophrenia and epilepsy (Pantazopoulos et al., 2010, 2015; Yutsudo and Kitagawa, 2015; Miyata and Kitagawa, 2016b). PNNs are markedly reduced in experimental ischemic stroke models (Härtig et al., 2016, 2017). Thus, it will be fascinating to assess the contribution of the molecular heterogeneity of PNN in models of these diseases.

## Author Contributions

SM and HK designed the research, analyzed the data, and wrote the manuscript. SM performed the experiments. MI produced ChGn-1 KO mice. HK coordinated the study. SM, SN, MI and HK reviewed the results and approved the final version of the manuscript.

## Conflict of Interest Statement

The authors declare that the research was conducted in the absence of any commercial or financial relationships that could be construed as a potential conflict of interest.
